# Hippocampal Malrotation Presenting With Treatment-Resistant Insomnia: A Case Report

**DOI:** 10.7759/cureus.46051

**Published:** 2023-09-27

**Authors:** Razan A Aljudibi, Asala A Albeladi, Salhah Alsulami, Wail Alamoudi

**Affiliations:** 1 College of Medicine, King Abdulaziz University Hospital, King Abdulaziz University, Jeddah, SAU; 2 Faculty of Medicine, King Abdulaziz University Hospital, King Abdulaziz University, Jeddah, SAU; 3 Internal Medicine, King Abdulaziz University Hospital, King Abdulaziz University, Jeddah, SAU

**Keywords:** seizure, sleep disorder, incomplete hippocampal inversion, hippocampal malrotation, chronic insomnia

## Abstract

A frequent complaint in medical settings is insomnia. Chronic insomnia is defined as the occurrence of sleep disturbance symptoms for a period of three months, three times per week, and in conjunction with at least one daytime symptom. In the case study, a young man with a documented seizure disorder underwent a thorough evaluation for chronic sleeplessness. Electroencephalograms, sleep investigations, and drug reviews were unsuccessful in determining the cause. Nonetheless, it was found that there was bilateral hippocampal malrotation. This link is distinct and hasn't been mentioned as a possible cause before.

## Introduction

Insomnia is one of the most common complaints encountered in medical practice, and it can affect up to 25% of adults [1]. According to the Diagnostic and Statistical Manual of Mental Disorders, Fifth Edition (DSM-5) criteria and the International Classification of Sleep Disorders, Third Edition (ICSD-3), chronic insomnia has been defined as the persistent symptoms of sleep disturbance for three months that present at least thre times per week in association with at least one daytime symptom [1]. Several pathophysiological mechanisms can underlie the development of insomnia. This includes misalignment of the circadian rhythm, dysfunction of the homoeostatic drive to sleep, reduction in GABAergic activity, overactivity of the orexinergic neurons, hyperarousal, as well as epigenetic mechanisms [2].

The hippocampus is involved in sleep control and emotions. [3] There are growing shreds of evidence that links severe insomnia with increased connectivity between the hippocampus and the left middle frontal gyrus. [4] Hippocampal malrotation (HIMAL) or incomplete hippocampal inversion is an atypical appearance of the hippocampus with abnormal medial location along the choroid fissure, a round or pyramidal shape, and/or collateral sulcus verticalization [5]. HIMAL has been linked to seizures and is considered an epileptogenic lesion [6]. Although changes in hippocampal volume have been described with chronic insomnia, the possible association of hippocampal abnormalities such as HIMAL with chronic insomnia has not been explored previously in the literature.

## Case presentation

**Figure 1:**
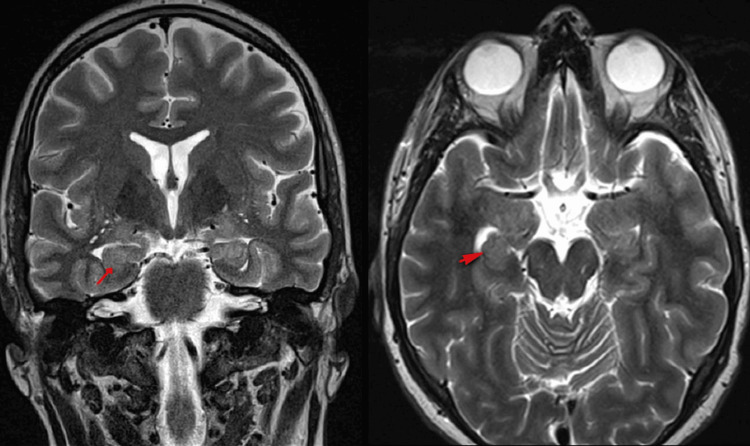
Seizure protocol an MRI with and without contrast was done. The axial and coronal T2-weighted images show a bilateral, rounded-shaped hippocampus with a loss of interdigitations likely related to hippocampal malrotations.

A 37-year-old businessman presented to the neurology clinic after having a witnessed tonic-clonic seizure that had been evaluated initially at the emergency department. He was previously medically healthy and was not on any regular medications. His interictal electroencephalogram did not find any epileptiform areas. He had a brain magnetic resonance imaging (MRI) scan that had no significant findings except for bilateral rounded hippocampal malrotation with preserved volume (Figure 1). He was started on Lamictal to prevent further seizure attacks, and he was referred to sleep medicine to manage insomnia and sleep deprivation, which may play a role in managing seizures as well.

At the sleep clinic, it was found that his insomnia started in his 20s, during his college years. He cannot recall any precipitating events. He had trouble falling asleep and continued to sleep after sleeping a couple of hours. His daytime symptoms were fatigue and memory problems. He reported that his sleep problem affected his grades back then. Over the years, his insomnia did not change, but it was less of a problem because working as a business owner gave him the pleasure of working at his own pace and times. He had no regular wake-up time, and he usually goes to bed between 11:00 p.m. and midnight. Despite his room being dark and quiet and his bed being comfortable, it usually took him more than 30 minutes to fall asleep and sleep for three to four hours before waking up and trying to force himself to sleep until the morning. He snores on occasion but denies any leg pain, numbness, headaches, shortness of breath, apnea, heartburn, or nocturia. Any episodes of sleep paralysis or cataplexy. During the day, he takes no naps and does not use caffeine or exercise regularly. Weekends and holidays have an insignificant effect on his sleeping patterns. He had never used melatonin or any other sleep aid. He was single at the time of the evaluation after an unsuccessful marriage last year, but he denies that it had any effect on his sleep. He denied any financial or other social issues. He denied any depression, suicidal ideation, or anxiety. He doesn’t smoke, consume alcohol, or use recreational substances. His objective data included a low score of 5/24 in the Epworth Sleepiness Scale and a STOP-Bang score of 3/8, indicating an intermediate risk for obstructive sleep apnea (OSA). The insomnia severity index questionnaire was not completed. His BMI was 26, and his neck circumference was 13.5 inches. His general blood work results were within normal ranges. Which included a complete blood count, kidney, and liver functions. A polysomnogram (PSG) was ordered to rule out any other coexisting sleep disorders that could worsen his insomnia. It was determined that the patient had mild obstructive sleep apnea with a positional component. His Apnea Hypopnea Index (AHI) was normalized on a second confirmatory PSG done with a non-supine sleeping protocol. The AHI is used to determine the severity of OSA. Both studies found poor sleep efficiency, decreased total sleep time, and early morning awakenings. Positional therapy as well as positive airway pressure (PAP) therapy were both ineffective in improving his sleep quality, insomnia, or daytime symptoms.

The patient was advised to practice good sleep hygiene, relaxation techniques, stimulus control, and sleep restriction. Those are some techniques used in cognitive-behavioral therapy for insomnia. We also discussed the option of short-term use of sleeping aid medications, but he preferred not to take any because of the potential side effects. In a later follow-up, his sleep complaints did not change much, and he complained of worsening memory, which started to interfere with his work and stress him out.

## Discussion

We present a case of chronic insomnia despite extensive workup for concomitant pathologies and failure of multiple treatment modalities. The only abnormal finding was HIMAL. Though mild OSA was found during the workup, therapy did not help to improve his sleep. We believe that his mild positional OSA did not affect his insomnia. Also, we have no reason to believe that his new-onset seizure, which was well-controlled, and being on antiepileptic treatment had any role in his insomnia because his sleep complaints did not change or worsen. Lamotrigine, on the other hand, is a newer-generation anti-epileptic drug that has a favorable effect on the sleep cycle and results in less frequent sleep disruption, thus improving seizure control and decreasing insomnia in epileptic patients [7].

The underlying causes of chronic insomnia are not well understood. Moreover, neural mechanisms for insomnia are difficult to study. In this case, we are reporting that the patient’s sleep and chronic insomnia were extensively investigated by his caring physicians. Multiple modalities of treatment were tried. Yet his symptoms were persistent and chronic. We wonder if the bilateral hippocampal malrotation could be driving his chronic insomnia. In the literature, a study reported both structural and increased functional connectivity in the hippocampus subregions, including the right cornu ammonis 1 and caudal hippocampus, with the medial prefrontal cortex (MPFC), in patients with insomnia disorder. It increased gradually from control to short-term insomnia to long-term insomnia [8]. Furthermore, structural abnormalities of the hippocampus are possibly associated with a decreased quality of sleep. A recent study of chronic insomnia patients with hippocampal atrophy demonstrated a higher arousal index during sleep. Additionally, people with hippocampal atrophy and chronic insomnia are more likely to experience a decline in their cognitive performance, which negatively impacts their quality of life [9]. Another study revealed that the hippocampus size is inversely correlated with the severity of chronic insomnia, along with a decline in cognitive function [10]. In this case, we propose that malrotation of the hippocampus can be linked to chronic insomnia. Longitudinal studies are needed to compare the presence of chronic insomnia in patients with hippocampal malrotation to those in the control group. Additionally, new modalities, such as functional MRI, may be used to support the diagnosis of chronic insomnia and guide the development of treatment strategies. Many studies have found a promising neuroimaging marker that includes abnormal functional connectivity and a low amplitude of low frequency fluctuation in multiple cortical and subcortical areas of the brain among patients with chronic insomnia [11].

## Conclusions

This report introduces a novel association between bilateral hippocampal malrotation (HIMAL) and chronic insomnia in a young man. To our knowledge, there has been no mention of this association in the existing literature. Further studies are necessary to elucidate the role of HIMAL in the pathogenesis of central insomnia. Moreover, additional research is required to gain a more comprehensive understanding of insomnia and its various forms.

## References

[1] Sateia MJ (2014). International classification of sleep disorders-third edition: highlights and modifications. Chest.

[2] Zhao L (2018). Neurobiology of common sleep disorders. Jr Neur Neur Diso.

[3] Havekes R, Abel T (2017). The tired hippocampus: the molecular impact of sleep deprivation on hippocampal function. Curr Opin Neurobiol.

[4] Leerssen J, Wassing R, Ramautar JR (2019). Increased hippocampal-prefrontal functional connectivity in insomnia. Neurobiol Learn Mem.

[5] Barsi P, Kenéz J, Solymosi D (2000). Hippocampal malrotation with normal corpus callosum: a new entity?. Neuroradiology.

[6] Tsai MH, Vaughan DN, Perchyonok Y, Fitt GJ, Scheffer IE, Berkovic SF, Jackson GD (2016). Hippocampal malrotation is an anatomic variant and has no clinical significance in MRI-negative temporal lobe epilepsy. Epilepsia.

[7] Foldvary N, Perry M, Lee J, Dinner D, Morris HH (2001). The effects of lamotrigine on sleep in patients with epilepsy. Epilepsia.

[8] Ma X, Jiang G, Tian J, Liu M, Fang J, Xu Y, Song T (2021). Convergent and divergent functional connectivityalterations of hippocampal subregions between short-term and chronic insomnia disorder. Brain Imaging Behav.

[9] Koo DL, Shin JH, Lim JS, Seong JK, Joo EY (2017). Changes in subcortical shape and cognitive function in patients with chronic insomnia. Sleep Med.

[10] O'Byrne JN, Berman Rosa M, Gouin JP, Dang-Vu TT (2014). Neuroimaging findings in primary insomnia. Pathol Biol (Paris).

[11] Yang N, Yuan S, Li C (2023). Diagnostic identification of chronic insomnia using ALFF and FC features of resting-state functional MRI and logistic regression approach. Sci Rep.

